# The Impact of Greenspace on Thermal Comfort in a Residential Quarter of Beijing, China

**DOI:** 10.3390/ijerph13121217

**Published:** 2016-12-08

**Authors:** Zhifeng Wu, Fanhua Kong, Yening Wang, Ranhao Sun, Liding Chen

**Affiliations:** 1 State Key Laboratory of Urban and Regional Ecology, Research Center for Eco-Environmental Sciences, Chinese Academy of Sciences, Beijing 100085, China; iamwuzf@163.com (Z.W.); wangyening0@163.com (Y.W.); rhsun@rcees.ac.cn (R.S.); 2 University of Chinese Academy of Sciences, Beijing 100049, China; 3 International Institute for Earth System Science, Nanjing University, Nanjing 210023, China; fanhuakong@163.com

**Keywords:** urban heat island, residential quarter, LAD, cooling effects of vegetation, thermal stress, ENVI-met, UTCI

## Abstract

With the process of urbanization, a large number of residential quarters, which is the main dwelling form in the urban area of Beijing, have been developed in last three decades to accommodate the rising population. In the context of intensification of urban heat island (UHI), the potential degradation of the thermal environment of residential quarters can give rise to a variety of problems affecting inhabitants’ health. This paper reports the results of a numerical study of the thermal conditions of a residential quarter on a typical summertime day under four greening modification scenarios, characterized by different leaf area density (LAD) profiles. The modelling results demonstrated that vegetation could evidently reduce near-surface air temperature, with the combination of grass and mature trees achieving as much as 1.5 °C of air temperature decrease compared with the non-green scenario. Vegetation can also lead to smaller air temperature fluctuations, which contribute to a more stable microclimate. The Universal Thermal Climate Index (UTCI) was then calculated to represent the variation of thermal environment of the study area. While grass is helpful in improving outdoor thermal comfort, trees are more effective in reducing the duration and expansion of suffering from severe heat stress. The results of this study showed that proper maintenance of vegetation, especially trees, is significant to improving the outdoor thermal environment in the summer season. In consideration of the deficiency of the current code in the management of greenspace in residential areas, we hope the results reported here will help promote the improvement of the code and related regulations for greenspace management.

## 1. Introduction

### 1.1. Micro-Scale Studies on the Urban Thermal Environment

Urbanization negatively affects the thermal environment, mainly due to the progressive replacement of natural surfaces with artificial alternatives. Such surface transition plus massive anthropogenic heat emitted from the cooling and heating of buildings, manufacturing, and transportation, result in the atmospheric urban heat island (UHI) phenomenon [1]. The increased urban air temperature seriously affects the energy consumption of buildings for cooling purposes, intensifies pollutant concentration, and causes detrimental effects on human health [2].

The outdoor thermal comfort can be investigated by field measurements or numerical models. In consideration of the large number of variables that exist in a typical urban block or neighborhood, field measurements have limited applicability and a growing number of numerical models have thus been developed. Various micro-scale models such as CTTC [3], Hoyano [4], MUST [5], SOLENE [6], RayMan [7], ENVI-met [8], etc. offer diverse opportunities to explore the microclimate variation of outdoor space from multiple aspects. Shashua-Bar et al. studied the thermal effect of built-up morphology and tree species with different canopy characteristics on microclimate formation [9]. Skelhorn et al. investigated the impact of vegetation types on air and surfaces temperature in Manchester, UK and the results showed that a 5% increase in mature deciduous trees can reduce mean hourly surface temperature by 1 °C over the course of a summer’s day [10]. Taleghani et al. studied the difference in outdoor thermal comfort level within five urban forms in Netherlands and the results showed that the courtyard provides the most comfortable microclimate in June compared to the other urban forms [11]. Although a large number of numerical models are documented, we must note that these models vary considerably depending on the scale, temporal and spatial resolution, and physical processes [10], and each model has its own limitations. Appropriate selection of model is a necessary fundamental step for accurate output.

The outdoor spaces in the residential area are one of the important public places for citizens to participate in social and recreational activities and the quality of the outdoor environment directly influences the livability of a residential quarter [12]. Given the growing interest in outdoor thermal comfort and the well-being of urban residents, a number of studies have been conducted to investigate the thermal comfort level of outdoor spaces of residential quarters in different climate regions [13,14,15,16,17]. Green vegetation has been proved to be a useful approach for improving the thermal environment at the neighborhood scale by increasing latent heat fluxes through evapotranspiration and reducing net heat storage though sheltering the direct solar radiation [18]. With the combination of building sizes and layout in relation to different types and configuration of greenspace, a residential quarter can develop diversified outdoor microenvironments [13,14]. Here, we conducted a study integrating numerical modeling with in-situ measurements to explore the effects of greenspace on variation of outdoor thermal environment of a typical high-rise residential quarter.

### 1.2. Context of Urban and Residential Development of Beijing

Beijing has undergone unprecedented urbanization over the last 30 years. The urban area of Beijing expanded from 801 km^2^ in 1980 to 2452 km^2^ in 2010 with an average growth rate of 3.7% [19]. The population in Beijing increased sharply from 8.7 million in 1978 to 21.1 million in 2013 [20], and the population is predicted to be well over 25 million by 2020. The ongoing urbanization in Beijing has placed great pressure on the urban thermal environment [14]. Current UHI intensity in Beijing is as high as 4 °C in summer [21].

In order to accommodate more people in the city, although controversy exists about the environmental sustainability of high-rise buildings in terms of energy consumption and social implication, this high-rise building development type has gradually become the mainstream for residential construction in Beijing [22]. One of the major advantages of high-rise buildings is their intensive use of urban land. High-rise buildings, especially high-rise tower buildings, have attracted great attention from property developers due to their higher profits. With gradually relaxation of housing policies since the Reform and Opening-up in 1978, especially after the abolition of the welfare housing distribution policy in the mid-1990s, the real estate market experienced a rapid development and a large number of high-rise buildings were built in Beijing [23]. A typical high-rise residential quarter in Beijing accommodates 10,000–15,000 inhabitants. These residents, especially the children and elderly, tend to spend a majority of their leisure time in the residential quarter. Some studies have shown that the ageing population is among the most vulnerable group to heat waves and elevated temperatures [24]. Thus, it is important to create a favorable microclimate for the sake of the residents’ health. However, the thermal conditions of a typical high-rise residential quarter in Beijing remain largely untouched. A relevant study becomes imperative in the context of worsening of UHI effects and extreme climate events, which has been predicted to increase in number, duration, and frequency with continuous global climate change [25].

To regulate the construction of high quality residential environments, related standards have been released in recent years [26,27]. The current Chinese national norm specifies permitted minimum greenspace ratios of 30% and 25% in newly constructed and reconstructed residential communities, respectively. Furthermore, the norms also specify the general pattern of vegetation arrangement in the form of public green areas or greenbelts mainly to satisfy the residents’ need for relaxing and social activities [26].

The outdoor thermal environment is influenced by the building pattern that determines the surface shadowed area and wind environment, and shading and evapotranspiration effects of vegetation. After the construction completion, however, the improvement of microclimate is mainly realized by greenspace management, such as tree canopy pruning and vegetation type selection. For a particular residential quarter, greenspace management is a part of the services offered by the property management company hired by the residents’ committee. Right management of greenspace is beneficial to create and maintain favorable outdoor environment. In this study, with the help of a microclimate model, we simulated the thermal environments in the selected residential quarter under several greening modification scenarios to investigate how the greenspace management may impact the surrounding microclimate.

### 1.3. Research Objectives

Considering the limited knowledge of integrated effects of vegetation on mitigating thermal environment and the requirements on how to manage the greenspace to improve microclimatic environment in the residential area, the objectives of this study were: (1) to compare the difference of cooling potential among different greening modification strategies; (2) to understand the modification of thermal environment and the variation of heat stress in summer season within a residential quarter in Beijing associated with vegetation type and quantity. The modeling of the micro-environment of a typical high-rise residential quarter would help evaluate quantitatively the general characteristic of the thermal environment and provide suggestions on how to design more sustainable living environment for urban residents.

## 2. The Study Area and Methodology

### 2.1. Selection of High-Rise Residential Quarter for Case Study

**Figure 1:**
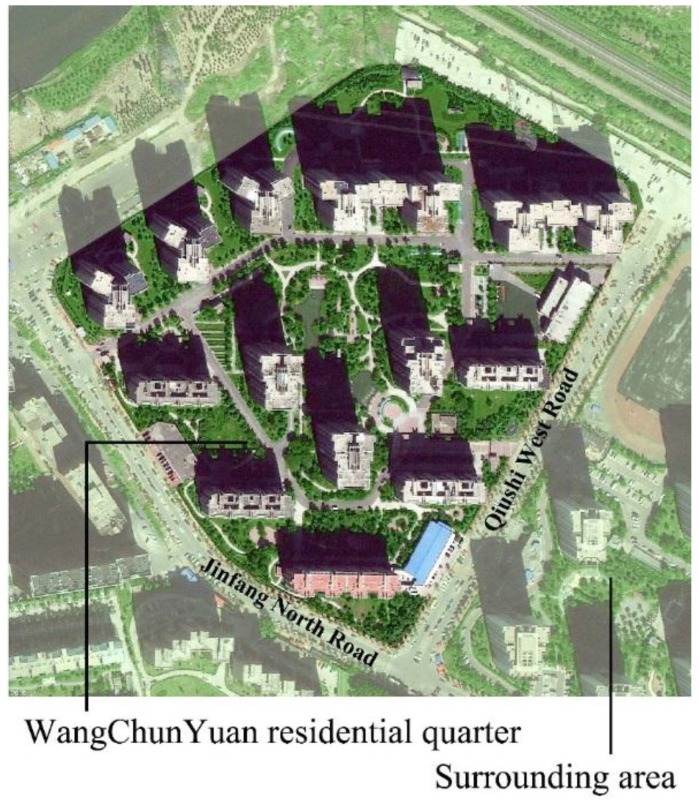
BaiduMap image of Wangchunyuan (WCY) residential quarter.

**Figure 2:**
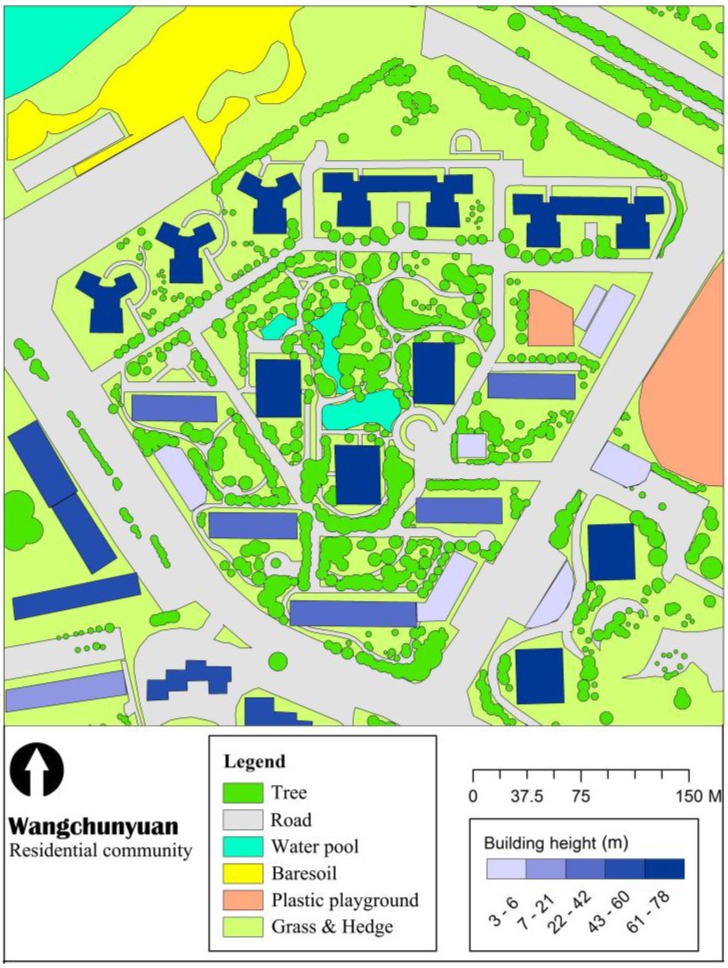
Land cover types of WCY and the surrounding area.

A typical high-rise residential quarter, known as Wangchunyuan (WCY) was selected for this study. WCY consists of eight buildings with 26 stories (78 m) and five buildings with 14 stories (42 m) (Figure 1). Inside the residential quarter, driveways were paved with asphalt, and sidewalks used concrete and cobblestone as pavement materials. The green area ratio is approximately 40%, which is above the average level of greenspace ratio for residential quarters in Beijing (Figure 2). There are approximately 2760 households in this residential quarter. According to a random house survey, the proportion of children (younger than 10 years old) and elderly (older than 60 years old) are 14% and 16%, respectively.

### 2.2. Microclimate Simulation Model

While empirical studies of the urban thermal environment have provided a growing understanding of variation in microclimate due to urban development, their applicability is sometimes limited because of the high complexity of urban environment. These limitations have then led to the development of various numerical models [28], which greatly facilitate urban environmental research [10].

In this study, the thermal environment of a residential quarter was studied with the help of ENVI-met 3.1 [8,29]. ENVI-met 3.1 is a three-dimensional and non-hydrostatic prognostic numerical model with computational fluid dynamics (CFD) as its core process. It can simulate the surface-plant-air interactions with respect to shortwave radiation fluxes from the sun to artificial surfaces and vegetation, longwave radiation from surfaces back to the sky and latent heat fluxes from vegetation into the ambient air. ENVI-met has a spatial resolution of 0.5–10 m and a temporal resolution of 10 s, which is suitable for microclimate studies on a neighborhood scale. ENVI-met has a large number of output parameters, including meteorological parameters such as air temperature, surface temperature, relative humidity, wind speed and direction, and thermal comfort indices such as mean radiant temperature and predicted mean vote (PMV).

Although ENVI-met has been extensively employed to examine the thermal environment, it has several limitations that must be noted: (1) the model does not take heat storage of building façades into account in the energy balance, which could result in an underestimation of the surface temperature of walls and ambient air temperature near the buildings; (2) it is not possible to create complicated building geometries such as curved shapes and shading devices within the model [30]; (3) the model does not incorporate the forcing of weather variables after initialization [10].

### 2.3. Simulation Preparation

The ENVI-met model requires two user-defined input files. The area input file is a 3-D representation of the area of interest allowing the user to design the layout of buildings, vegetation, artificial surfaces and soils. The configuration file contains meteorological parameters and surface properties for initialization of the model and computation of energy exchange.

To represent the configuration of buildings and greenspace of WCY as precisely as possible, we defined the area input file combining field measurements and high-resolution BaiduMap image. We then produced 3-D area input files with 2 m and 5 m horizontal and vertical resolution, respectively, with 235 × 235 × 25 grids for the whole modeled area. In addition to the WCY residential quarter area, the surrounding buildings and vegetation were also included in the model to create a more realistic environment. Furthermore, we added five nesting grids to each side of the model to increase the stability of the simulation for elements close to the border of the study area.

To obtain WCY’s meteorological data, a HOBO U30 weather station (Onset Computer Corp., Bourne, MA, USA) was placed in an open space in the center of a pocket park in WCY with >10 m away from surrounding trees and buildings. The HOBO U30 is an enhanced multi-sensor weather station. It measured a wide range of weather parameters, including wind speed and direction, air temperature, relative humidity, soil temperature and humidity at 2 m above ground level. These parameters are required for the ENVI-met simulation.

UHI phenomenon should be viewed from a multi-scale perspective [31]. Scale is a fundamental concept for understanding the ways by which urban surfaces interact with adjacent atmospheric layers [32]. The meteorological conditions at any locality are controlled by large scale atmospheric circulation and, at the same time, strongly influenced by the local environment, such as building layout and vegetation arrangement. Interactions between the two scales are often present, and it is difficult to distinguish which one is dominant [33]. Meteorological data collected on days with high atmospheric stability is preferable because under this weather system, the local environment is more likely to dominate the variation of meteorological conditions. Simulation results under stable atmospheric conditions tend to be more accurate and reflect more details of characteristics of study area. 11 August 2014 was selected for simulation because it has clear sky, weak winds and no precipitation in 3 days, which favor the generation of UHI. Due to the intensive disturbance to near-surface air flow caused by surrounding high-rise buildings and trees, the wind speed and direction observations at 2 m above ground were not actually representative of the local climate zone WCY is located in. We therefore placed an anemograph on the top of a 26-story building (76 m) to collect wind data with little disturbance from surrounding buildings and vegetation and then calculated the wind speed of the 10 m level according to the wind profile power law. We ran ENVI-met for a 24 h period, starting from 6:00, 11 August 2014. The main input parameters of the ENVI-met simulation are listed in Table 1.

In ENVI-met, the leaf area density (LAD) is an important variable related to multiple processes, such as solar interception, evapotranspiration, wind dragging, and additional atmospheric turbulence due to vegetation. LAD determines the size of the plant-atmospheric interfaces and thus plays a key role in the exchange of energy and mass between vegetation and atmosphere [34]. The ENVI-met model provided several inbuilt LAD profiles for different canopy structures. However, these LAD profiles were based on only a few reference profiles and therefore are not applicable universally [29]. To obtain more accurate simulation results, we established our own LAD profiles of the biomass of WCY.

**Figure 3:**
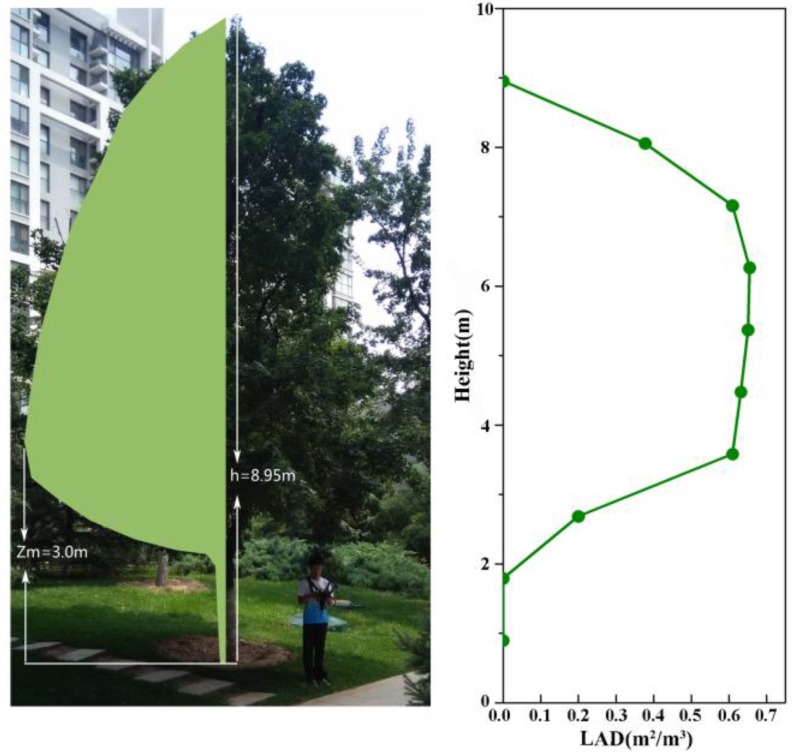
Photo of a mono maple (*Acer mono* Maxim) and its LADs versus height.

Leaf area index (LAI) can be assessed directly by using harvesting methods or non-harvesting litter traps during autumn’s leaf-fall period in deciduous forests. While the direct methods give the most accurate results, they are destructive and time-consuming. Some indirect methods and instruments, which are faster and amenable to automation, have been developed. After comparison of different instruments used for LAI assessment, LAI-2000 and hemispherical cameras were proved to be the most satisfactory methods [34]. The vegetation parameters required by ENVI-met for the WCY simulation were collected on-site. We measured the LAI using a LAI-2000 plant canopy analyzer (LI-COR Inc., Lincoln, NE, USA) for major tree species. Then the LADs of specific tree species were calculated according to the empirical LAD model provided by Lalic and Mihailovic [35]. Parameters required for this model include tree height (h), the maximum value of LAD (Lm), and the corresponding height with the maximum value of LAD (zm). Then, the calculated LADs were put in the ENVI-met plant database for 3-D reconstruction of the tree canopy. Figure 3 represented a mono maple (*Acer mono* Maxim) which is one of the most widespread deciduous trees planted in residential quarters in Beijing and its LADs at different height levels.

### 2.4. Development of Greening Modification Scenarios

Through the field survey in the selected residential quarters in Beijing, we found several reasons caused the vegetation degradation. One of the main reasons is that the improper management of the greenspace through communications and interactions with the residents or property manager of these residential quarters. The improper management typically included irregular irrigation, trampling without control, improper pruning, etc. The improper management problem is due to many reasons such as poor operations of the property management company which is responsible for the maintenance of greenspace, or the absence of greenspace management that often occurs in older residential communities.

According to the current vegetation conditions of residential quarters of Beijing, we developed four vegetation greening scenarios to understand the impacts of greening modifications on thermal environment in WCY (Figure 2). Apart from the base case (CS—Current Situation scenario) based on the existing greenspace configuration, the other three scenarios are: NV (No Vegetation scenario) designed to represent the most extreme non-green case; OG (Only Grass scenario) replaced all existing trees with grass; DL (Double LAD scenario) applied by doubling the LAD of existing trees to investigate the transform of outdoor environment brought by proper vegetation management.

### 2.5. Model Validation

Variation of ambient air temperature (T_a_) is controlled by different factors such as the thermal properties of surrounding natural and artificial surfaces, ventilation conditions, and environmental shading rate. It is reasonable to use T_a_ as a composite index to represent the thermal status of a specific site. This study focused on the period in which people more likely to participate in outdoor activities. We thus compared hourly T_a_ from 7:00 to 22:00 derived from meteorological station with mean modeled T_a_ throughout the whole model environment. Both the observed and predicted temperature peaked at 15:00 but with different rates of temperature change. The measured temperature increased 1.12 °C per hour during 6:00–15:00 and decreased 0.66 °C per hour after 15:00. Simulation results showed a lower rate of change in both the warming-up (0.86 °C/h) and cooling-down periods (−0.39 °C/h). Previous studies have reported that ENVI-met produced time-series temperatures with lower variance [18,36], which could be explained by inaccuracies in simulation input for surface thermos-physical properties and vegetation conditions.

**Figure 4:**
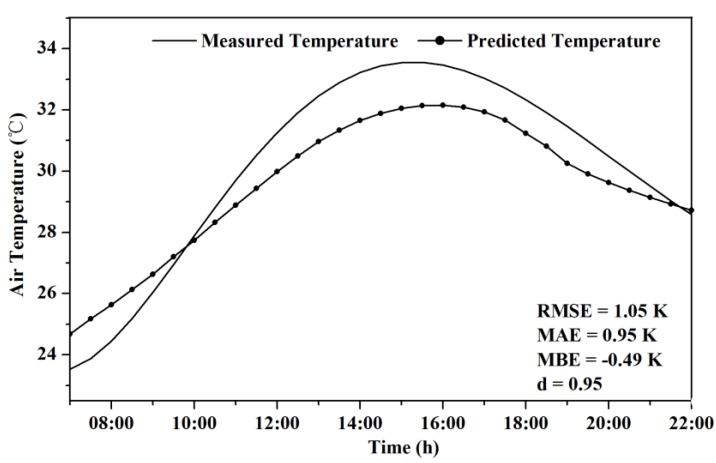
Comparison between hourly averaged air temperature from meteorological station and mean modeled temperature from ENVI-met simulations at 1.5 m above ground. RMSE = root mean squared error; MAE = mean absolute error; MBE = mean bias error; d = index of agreement.

The root mean square error (RMSE), mean average error (MAE), mean bias error (MBE) and the index of agreement (d) were calculated to evaluate the accuracy of the simulation results. According to results (Figure 4), the RMSE is 1.05 °C and MAE, MBE are 0.95 °C and −0.49 °C respectively. The index of agreement was 0.95, which indicates even with the lower variance and the underestimation of peak daytime temperature, modeled T_a_ at 1.5 m above ground showed generally good agreement with field measurements.

### 2.6. Calculation of Universal Thermal Climate Index (UTCI)

After decades of development, thermal biometeorology has advanced considerably with the development of heat budget models. Although these models are appropriate for use in any kind of assessment of the thermal environment, none of them is accepted as a fundamental standard, either by researchers or by end-users [37]. The International Society on Biometeorology recognized the shortcomings in existing thermal indices and developed the Universal Thermal Climate Index (UTCI) [38]. Blazejczyk et al. compared UTCI with several thermal indices including the heat index, the wet-bulb globe temperature, the standard effective temperature, PMV and the physiological equivalent temperature etc. based on datasets of global, regional and local scales. The results indicated that UTCI represented specific climates, weather and locations much better, and depicted temporal variability of thermal conditions better than the other indices [39].

According to the UTCI assessment scale provided by The Commission for Thermal Physiology of the International Union of Physiological Sciences (Table 2), we analyzed the variations of area exposed to different stress levels under four scenarios.

## 3. Results and Analysis

### 3.1. Effect of Vegetation on Air Temperature

**Figure 5:**
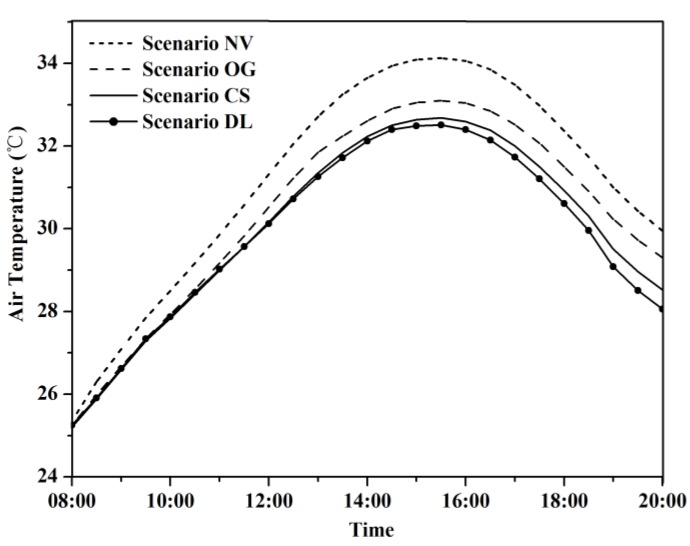
Comparison of average hourly air temperature at 1.5 m above ground. NV, OG, CS and DL are short for No Vegetation, Only Grass, Current Situation and Double LAD respectively.

**Figure 6:**
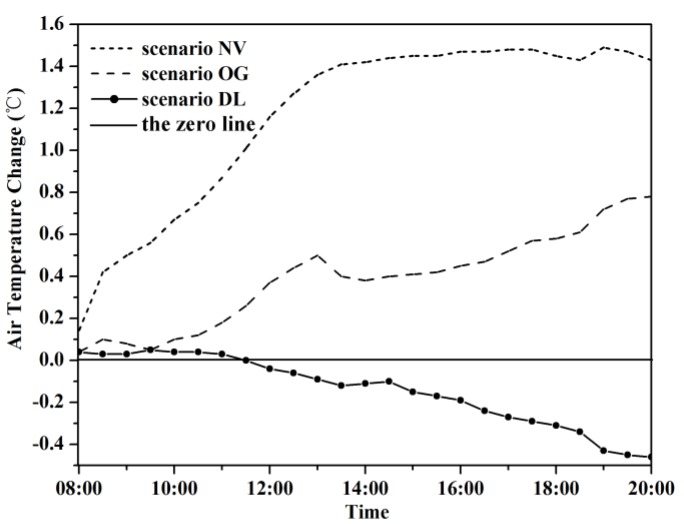
Air temperature difference of green modification scenarios compared with the base case at 1.5 m above ground. NV, OG and DL are short for No Vegetation, Only Grass and Double LAD respectively.

We chose hourly averaged air temperature at 1.5 m above ground over the whole modeled area to examine the thermal conditions under four greening modification scenarios. According to the ENVI-met simulation results, the air temperatures of the base case were between 23.9 °C and 32.6 °C during 8:00 to 20:00 with the peak temperature at 15:00. The base case was then compared with three other scenarios over the same period of time. Figure 5 shows the variations of air temperatures at 1.5 m above ground of four scenarios (NV, OG, CS, and DL), which follow similar pattern of constantly increasing from 8:00 to 15:00 and decreasing from 16:00 to 20:00. The scenario NV and scenario OG showed higher temperatures over the whole course of the timeframe, and the air temperature differences from the base case gradually increased from 8:00 onwards with the highest increasing of temperature (1.49 °C and 0.72 °C, respectively) recorded at 20:00 (Figure 6). In term of the vegetation, the results indicated that both grass and tree have the potential to reduce air temperature. Grass lowers air temperature mainly through evapotranspiration, while tree lowers air temperature by both evapotranspiration and sheltering solar irradiance from baking the area below the tree canopy. The scenario DL displayed marginal decrease in air temperature (0.17 °C on average) compared with the base case with the largest temperature decrease of 0.47 °C (Figure 6). When looking into particular spatial positions, the scenario DL demonstrated noticeably different cooling rates, which can achieve up to 2.4 °C right beneath the tree canopy, while the cooling power is negligible at positions outside the canopy and exposed directly to solar radiation.

**Figure 7:**
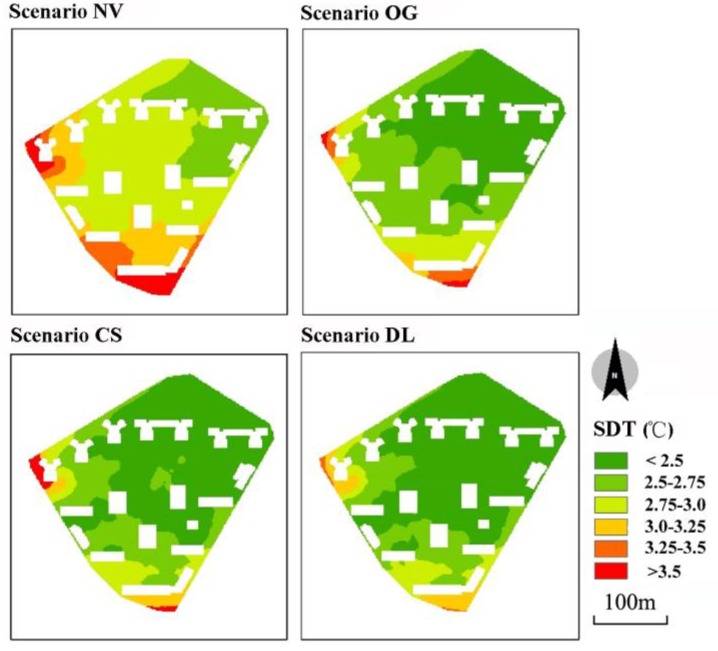
Standard deviation of air temperature at 1.5 m above ground at grid level from 8:00 to 20:00. SDT = standard deviation of air temperature. NV, OG, CS and DL are short for No Vegetation, Only Grass, Current Situation and Double LAD, respectively.

Standard deviation is a commonly used measure that quantifies the amount of variation or dispersion of a set of data values. Higher standard deviation indicates that the data points are spread out over a wider range. We used the spatial statistics tools in ArcGIS 9.3 (Esri Inc., Redlands, CA, USA) to calculate the standard deviation of air temperature (SDT) of each grid in modeled area during 8:00–20:00 to understand the influences from vegetation on air temperature (Figure 7). Scenario NV clearly showed higher SDT than other three scenarios. With the addition of vegetation, the area with highest SDT (>3.5 °C) gradually decreased and disappeared in scenario CS and DL, while the percentage of area with smallest SDT (<2.5 °C) expanded considerably from zero in scenario NV to 43%, 62% and 65% in scenario OG, CS and DL respectively. The results implied that in addition to lower air temperature, vegetation could also lead to a more stable thermal environment with smaller temperature variation.

### 3.2. Effect of Vegetation on Thermal Sensation

**Figure 8:**
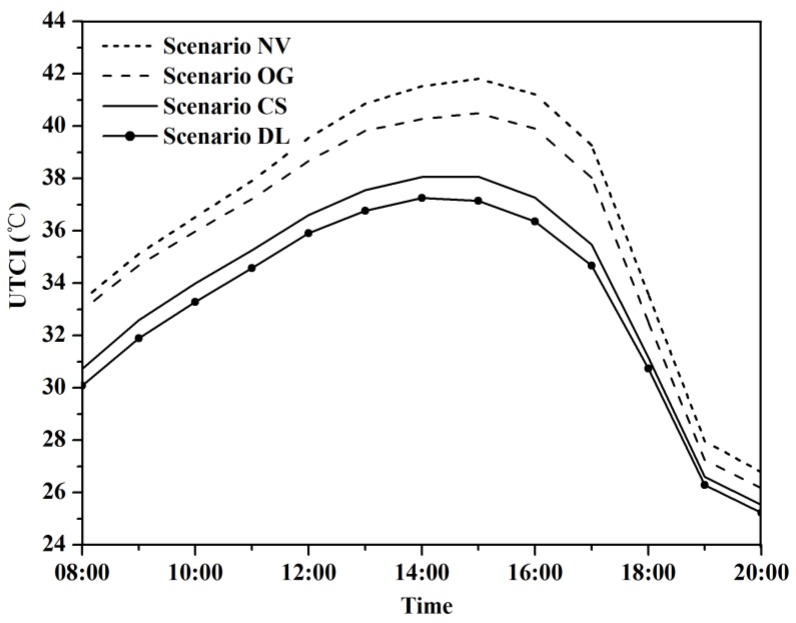
Comparison of average hourly UTCI. NV, OG, CS and DL are short for No Vegetation, Only Grass, Current Situation and Double LAD, respectively.

**Figure 9:**
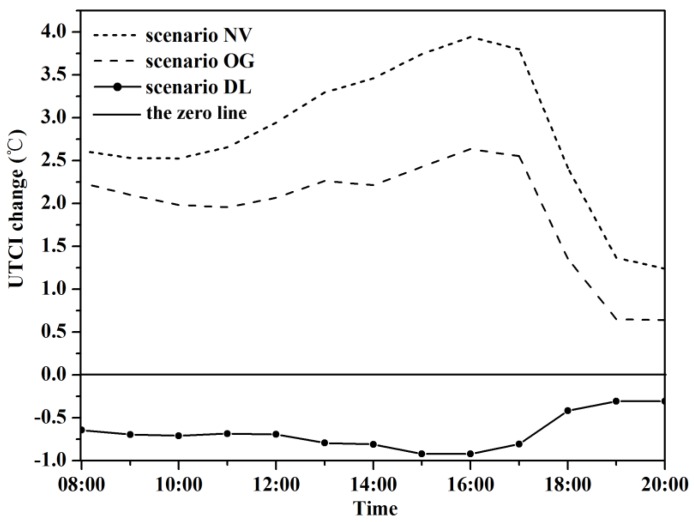
UTCI differences of greening modification scenarios from the base case. NV, OG and DL are short for No Vegetation, Only Grass and Double LAD, respectively.

We calculated UTCI using the outputs derived from ENVI-met to investigate the change of outdoor thermal comfort status of whole WCY (Figure 8) from 8:00 to 20:00. The UTCI of the CS (base case) varied from 19.87 °C to 45.58 °C, and scenario NV and OG showed 2.8 °C and 1.9 °C, respectively higher on average than the base case (Figure 9). When compared the base case with degradation scenarios on a grid level, the magnitude of cooling was notably larger than at the whole modelled area scale due to the shading effects of trees, which can provide an approximately 10 °C cooler thermal environment in contrast with the bare soil situation in scenario NV. Trees with denser canopy can intercept more solar radiation and mitigate the thermal stress during summer daytime, which is supported by our results that scenario DL was 0.67 °C cooler on average than the base case.

**Figure 10:**
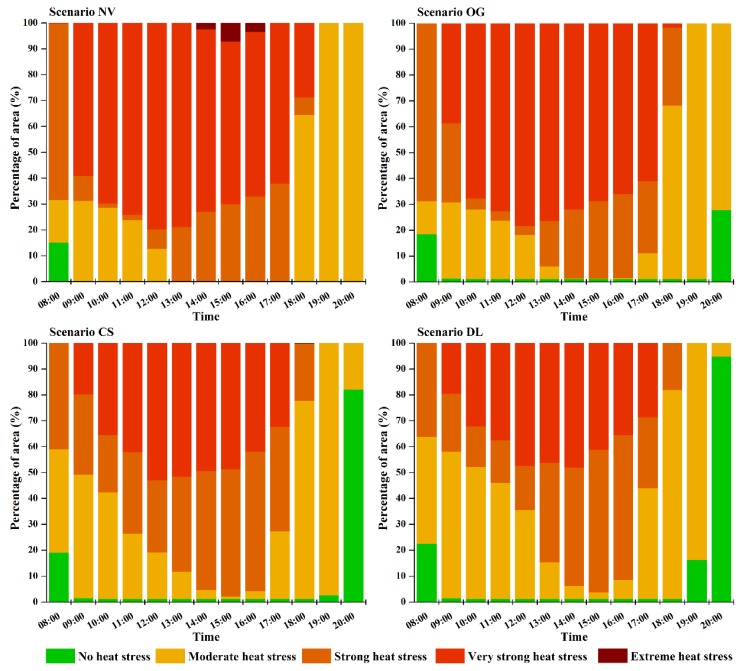
Variation of area experiencing different heat stress levels under 4 developed greening scenarios. NV, OG, CS and DL are short for No Vegetation, Only Grass, Current Situation and Double LAD, respectively.

From the base case, it is explicitly shown that almost the whole WCY was under thermal stress of different levels from 9:00 to 19:00. Areas with no heat stress only exist at morning time in a small proportion (19.2%) and after the sunset (approximately at 19:15). The results demonstrated that during the summer day, people can only enjoy outdoor spaces of WCY before 9:00 and during the evening hours after 19:00. Compared with the base case, the areas of scenario NV and OG that suffered from strong thermal stress were reduced during 10:00 to 16:00, while the area exposed to more severe heat stress level (very strong heat stress) showed a noticeable increase from 8:00 to 19:00, which indicated that the outdoor thermal environment was deteriorating. For scenario NV, a small area of WCY even experienced extreme thermal stress during the period of 14:00 to 16:00 (Figure 10). This illustrated that the number of hours and area of the studied quarter that can be enjoyed by residents were further reduced in the two degradation scenarios. Doubling trees LAD profiles resulted in a decrease in area suffered from strong thermal stress and very strong thermal stress and an increase in area under no thermal stress and moderate thermal stress, indicating tree played an important role in mitigating thermal environment due to its denser canopy, which could shelter more solar radiation from ground surfaces.

## 4. Discussion

### 4.1. Issues about the Microclimate Modelling

Although there are still several limitations, such as underestimation of daytime air temperature, ENVI-met produced accurate enough results to conduct an outdoor thermal environment evaluation. The simulation process indicates that more attention needs to be paid to LAD, which is an important parameter of vegetation for characterizing different vegetation types. The LAD profiles provided by ENVI-met are, according to the developer of the model, “rather handmade and based on only a few reference profiles”. Hence, the default vegetation information is not universally practical, especially for regions with different climatic characteristics. A specific vegetation database should be built to represent local vegetation by a vegetation survey rather than simply utilizing the data provided by the model.

Even though ENVI-met has already considerably simplified the procedure of the simulation of thermal environment compared with CFD, it is still time consuming and impractical for urban planning. Simpler models or indices with reliable predictions of the thermal environment or wind environment of interest area are required. Future work should pay more attention to the development of the model and indices.

### 4.2. Implications of Vegetation on Mitigating Microclimate

Previous studies have indicated that synoptic conditions have significant impact on the development and characteristics of urban heat island, which is better developed under clear, calm conditions [40,41]. One major adverse impact of urban heat island phenomenon is the increase of human discomfort. In the current study, we selected a typical summer day to study the regulation effect of greenspace on outdoor thermal environment. Four greening modification scenarios were developed to investigate the cooling effect of the types and amounts of greenspaces. The average air temperature of the four scenarios during the period of 8:00 to 20:00 were 31.2 °C, 30.4 °C, 30.0 °C and 29.8 °C, respectively, with the maximum cooling power of 1.92 °C comparing scenario DL with scenario NV. The results were consistent with many previous studies that indicated vegetation is an important consideration for improving the outdoor thermal environment. Meanwhile, with the increase of vegetation, the air temperature variation became smaller. The effects of vegetation on the microclimate are mainly due to its shading effects that help ground surfaces absorb less radiation and its evapotranspiration process that cools the ambient air [42]. In summary, increasing vegetation not only led to a cooler environment but also helped to form a more stable meteorological condition that is potentially beneficial to susceptible populations such as the elderly and children.

Trees are essential not only in reducing near-surface air temperatures in residential communities but also in improving outdoor thermal comfort through providing shade for pedestrians. Trees with dense canopy act as an umbrella that intercepts a large amount of solar energy from heating the ground surface and keeping the space relatively comfortable in term of thermal perception. According to the results, scenarios with trees (scenarios CS and DL) significantly reduced the area suffering from very strong heat stress comparing with scenarios without trees (scenarios NV and OG). This implies that trees behaved in a more advantageous way in contrast to bare soil and grass in mitigating local thermal environment and providing relatively comfortable outdoor spaces. To offset the high cost of planting and maintenance of trees, some constructors of residential quarters chose to plant more grass to achieve the specified greening standards. However, the grass, apparently cannot replace the role trees playing in improving the thermal environment.

It was found that more vegetation could result in a cooler environment and smaller air temperature variations. However, it might not be the best choice to replace all greenspace with trees in term of mitigating thermal environment. Open spaces covered with grass or sparse trees are also advisable for recreational activities after sunset due to their higher cooling rate. The vegetation arrangement deserve further investigation at the neighborhood scale.

Many previous studies focused on particular times of the day to analyze the thermal performance of vegetation on studied area, while looking at the whole picture of thermal status from a continuous period of time would be more meaningful to help residents to reasonably plan their time for outdoor activities. As our results showed, though vegetation, especially trees, improved the thermal comfort level in WCY, almost all the daytime experienced heat stress of various levels. Only some areas experienced no heat stress before 9:00 and after 19:00. Actually, by appropriate protective measures, moderate heat stress would cause no harm to people’s health, which provided residents more time and spaces engaging in outdoor activities. It should be noted that 13:00–17:00 was the worst period when more than 80% of outdoor spaces of WCY suffered from strong heat stress and very strong heat for all four scenarios, and a reduction of physical activity is strongly suggested to lower risk of thermal disorder. It is clear that only vegetation is not enough to obtain thermally comfortable conditions, especially on summer days. Multiple mitigating strategies such as sheltering devices, water pools, would be more helpful in beating the heat.

The present study focused on high-rise residential quarter due to this development type is the mainstream for residential construction. Meanwhile, a large number of middle-rise and low-rise neighborhoods exist in Beijing. Stewart and Oke developed a classification system named “Local Climate Zones-LCZs” to address the inconsistency of methods and communications in heat island studies [43]. The classification divides urban and rural landscapes into 17 standard classes, with each characterized by unique surface structure (building/tree height and spacing), land cover characteristics (impervious fraction, pavement albedo and thermal admittance) [44]. Several studies have analyzed the microclimate of different LCZs by green modification and confirmed the vegetation, especially trees, could moderate microclimate and improve thermal comfort level [10,17,45,46,47]. For example, Middel et al. simulated nine combined tree planting and landscaping scenarios at a low-rise neighborhood using ENVI-met and the results showed that the relationship between percent canopy cover and air temperature reduction was linear, with an increase in tree canopy cover from 10% to 25% resulting in an average daytime cooling rate of 2.0 °C [46]. With the increase of height and density, buildings have a strong influence on the near surface thermal environment by casting shadows and surface roughness modification [48]. This cooling rate is apparently higher than our results, which might partly due to the local climatic characteristics, and the other reason might be stronger control effect of higher buildings over the microclimate. That means the same amount of greenspace might exert different cooling effect in different LCZs. More studies should be conducted to explore the cooling power of greenspace under various building environment.

The code for planning and design on urban residential areas was initially implemented in February 1994 and undergone partial modification in 2002. With the rapid development of the economy and the considerable the improvement of living standards in China, residents’ requirements for the residential environment are raising. The amelioration of the outdoor thermal environment is one aspect of environment improvement. However, the code only sets out several rules on greenspace type and greening rate. There is no items involving vegetation type and vegetation configuration. Some residential quarters which are up to the greening rate standard of this code have only scattered trees and the major part of the land was planted with grass. Our research demonstrated the importance of trees and their maintenance on the mitigation of thermal environment, which should be paid more attention in greenspace management in residential area.

## 5. Conclusions

This paper reports the results of microclimatic modelling of the effects of different greening modification scenarios on ambient air temperature and outdoor thermal comfort level in a typical residential quarter of a typical summertime day in Beijing, China. The results displayed that the combination of grass and tree can lead to a maximum reduction of approximately 2 °C in air temperature compared with scenarios of no vegetation. Trees play an important role in improving the outdoor comfort level due to their shading effects protecting people from being directly exposed in solar radiation. In consideration of the deficiencies of the current code for urban residential areas planning and design, we hope the results reported here will help to promote the improvement of greenspace management regulations. Also, it has implications for greenspace maintenance, highlighting the potential benefits provided by vegetation in good condition. Moreover, though high-rise buildings are considered as environmentally sustainable due to their intensive usage of land, vegetation is proved to be an indispensable part in mitigating the near-surface thermal environment, especially during heat wave periods.

## Figures and Tables

**Table 1:** The main input parameters of the ENVI-met simulation.

Items	User Input during Simulations
Simulation day	11 August 2014
Simulation time	24 h
Initial temperature	24.5 °C
Relative humidity 2 m aboveground	45%
Wind speed	1.5 m/s
Wind direction	220°
Indoor temperature	19 °C
Heat transmission of walls/roofs	1.7/2.0 W/m^2^ respectively
Albedo of walls/roofs	0.3/0.4 respectively

**Table 2:** UTCI range and corresponding thermal stress level.

UTCI (°C) Range	Stress Category	Protective Measures
+9 to +26	No thermal stress	Physiological thermoregulation is sufficient to maintain thermal comfort
+26 to +32	Moderate heat stress	Drinking > 0.35 L/h of fluids is necessary
+32 to +38	Strong heat stress	Drinking > 0.35 L/h of fluids is necessary. Staying in shaded places is recommended. Periodically, a reduction in physical activity is recommended
+38 to +46	Very strong heat stress	Temporary use of air-conditioned rooms or staying or staying in shaded places is periodically necessary. Drinking > 0.5 L/h is necessary. Reduce physical activity
>46	Extreme heat stress	Temporary body cooling is periodically necessary. Drinking > 0.5 L/h is necessary. Avoid physical activity
