# Erratum: Wu, Z., et al. The Impact of Greenspace on Thermal Comfort in a Residential Quarter of Beijing, China. *Int. J. Environ. Res. Public Health* 2016, *13*, 1217

**DOI:** 10.3390/ijerph14020156

**Published:** 2017-02-07

**Authors:** International Journal of Environmental Research, Public Health Editorial Office

**Affiliations:** MDPI AG, St. Alban-Anlage 66, 4052 Basel, Switzerland; ijerph@mdpi.com; Tel.: +41-61-683-77-34

Due to an error during production, the project numbers of the National Natural Science Foundation of China projects in the Acknowledgments Section of the published paper [1] were incorrect. The correct project numbers should be 41230633 and 41471150.

We apologize for any inconvenience caused to the readers or authors by this error. The article will be updated and the original will remain on the article webpage.
